# A high-throughput molecular data resource for cutaneous neurofibromas

**DOI:** 10.1038/sdata.2017.45

**Published:** 2017-04-11

**Authors:** Sara J.C. Gosline, Hubert Weinberg, Pamela Knight, Thomas Yu, Xindi Guo, Nripesh Prasad, Angela Jones, Shristi Shrestha, Braden Boone, Shawn E. Levy, Salvatore La Rosa, Justin Guinney, Annette Bakker

**Affiliations:** 1 Computational Oncology, Sage Bionetworks, Seattle, Washington 98109, USA; 2 Plastic and Reconstructive Surgery, Mount Sinai Medical Center, New York, New York 10028, USA; 3 Children’s Tumour Foundation, New York, New York 10005, USA; 4 Hudson-Alpha, Huntsville, Alabama 35806, USA

**Keywords:** Paediatric cancer, RNA sequencing, Personalized medicine, Genotyping and haplotyping, DNA sequencing

## Abstract

Neurofibromatosis type 1 (NF1) is a genetic disorder with a range of clinical manifestations such as widespread growth of benign tumours called neurofibromas, pain, learning disorders, bone deformities, vascular abnormalities and even malignant tumours. With the establishment of the Children’s Tumour Foundation biobank, neurofibroma samples can now be collected directly from patients to be analysed by the larger scientific community. This work describes a pilot study to characterize one class of neurofibroma, cutaneous neurofibromas, by molecularly profiling of ~40 cutaneous neurofibromas collected from 11 individual patients. Data collected from each tumour includes (1) SNP Arrays, (2) Whole genome sequencing (WGS) and (3) RNA-Sequencing. These data are now freely available for further analysis at http://www.synapse.org/cutaneousNF.

## Background & Summary

Neurofibromatosis (NF) describes three forms of genetic conditions: NF1, NF2 and Schwannomatosis. People with NF may exhibit a wide range of clinical symptoms including neurocognitive deficits, brain tumours, bone abnormalities, and vascular disease. Effective treatments for people with NF are lacking due to the diversity of disease symptoms and underlying difficulty in treating any genetic disorder involving the loss of a tumour suppressor genes.

NF1 is the most common of the NF syndromes with an incidence of approximately 1/2,500 worldwide^1^. While NF1 has been linked to loss of function in the *NF1* gene—a known tumour suppressor—due to mutation or deletion, there is a high degree of phenotypic diversity in the patient population, making it difficult to predict disease progression and to treat it effectively^2^. NF1 patients are susceptible to growths of two types of neurofibromas: plexiform neurofibromas, that can cause pain in affected patients and can transform into deadly malignant peripheral nerve sheath tumours, and cutaneous neurofibromas.

Cutaneous neurofibromas (cNFs) are benign lesions that occur during adolescence in NF1 patients and increase throughout their lives. Cutaneous neurofibromas can be painful and disfiguring although they generally do not affect the overall physical health of the affected individual. These lesions generally arise upon mutation or loss of the second *NF1* (ref. 3) allele. Although the microenvironment has been known to play a role in tumour growth^4,5^, little is known about the molecular aetiology of these tumours themselves. To address this knowledge gap in the cutaneous neurofibroma field, we sought to improve overall knowledge of cutaneous neurofibromas through high-throughput molecular characterization of a diverse set of samples across patients.

**Figure 1:**
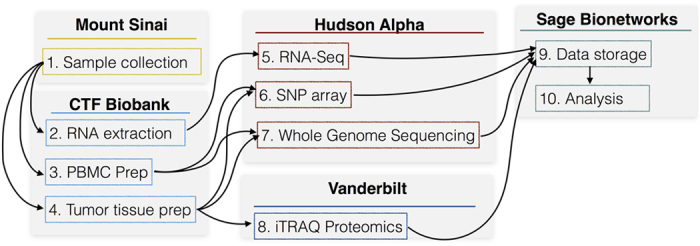
From sample to dataset. A description of the collaborative effort that enabled the Cutaneous NF data repository and subsequent analysis of the samples.

This resource represents a highly collaborative effort that spans multiple institutions (see Fig. 1). cNF samples and patient blood were collected in the clinic by Children’s Tumour Foundation (CTF), who preserved and annotated the samples and sent them to the CTF Biobank. DNA from tumour and blood was profiled by (1) whole genome sequencing (WGS) and (2) Single nucleotide polymorphism (SNP) array and tumour RNA was profiled by (3) RNA-Sequencing. Data was then annotated and compiled into a single resource built on the Synapse^6^ platform available at http://www.synapse.org/cutaneousNF (Data Citation 1).

## Methods

### Sample collection

Eligible patients between the ages of 18 and 65 who were scheduled to receive elective surgery to remove cutaneous neurofibromas were invited to donate surgical discards as well as blood and urine samples. Patients consented to the release of their tissue samples and any resultant data through a protocol approved by Western IRB (Puyallup, WA). Patient-reported medical, family and NF1 history was also collected. Sample information is available in the table in the data repository (syn5556216; Data Citation 1).

Each tumour was sub-divided with each part either preserved in formalin, flash frozen in liquid nitrogen, or placed in RNA later solution within 45 min of removal from the patient. If there was not enough tissue for all three methods of preservation, priority was given to formalin, followed by frozen. Samples noted by the patient as recently growing were annotated as such, and each sample was labelled by the location of the body from which it was retrieved. Skin covering the tumours was not removed prior to preservation but was removed prior to sequencing.

30 cc of blood was also collected from each patient and stored at −80 °C. PBMCs were isolated and stored at −150 °C.

### Whole genome sequencing

Genomic DNA (gDNA) was extracted from both frozen tumour and blood tissues using the DNeasy mini kit (Qiagen, Valencia, CA, USA) according to the manufacturer's protocol. The final elution was performed in 50 μl of RNase-free sterile distilled water. The concentration of the gDNA was estimated using the Qubit 2.0 Fluorometer (Invitrogen); samples with less than 500 ng were discarded. The integrity of the gDNA was assessed by running an aliquot of the gDNA on 0–8% agarose gel to confirm absence of RNA or protein bands as well as absence of a smear that would indicate degradation. One microgram gDNA was required for downstream whole genome library preparation applications. The samples were then sonicated on the Covaris LE220 (Covaris Inc, Woburn, MA, USA) to achieve an average target size of 400 bp. QC analysis of the post-sonicated material was done using Caliper LabChip GX (Perkin Elmer, Hopkinton, MA, USA).

Standard whole genome library prep was done using the NEBNext DNA Library Prep Reagent Set for Illumina (New England BioLabs Inc., Ipswich, MA, USA) as per manufacturer's recommended protocol. Library quality was assessed using the Qubit 2.0 Fluorometer, and the library concentration was estimated by utilizing a DNA 1,000 Chip on an Agilent 2,100 Bioanalyzer. Accurate quantification for sequencing applications was determined using the qPCR-based KAPA Biosystems Library Quantification Kit (Kapa Biosystems, Inc., Woburn, MA, USA). Each sample was then sequenced on an individual lane on an Illumina HiSeqX sequencer (Illumina, Inc., San Diego, CA, USA).

Reads were mapped to the genome and variants identified using the Dragen^7^ program with default settings. The resulting VCF files (syn5522788; Data Citation 1) were then sorted and updated to remove errors and analysed using the GATK pipeline^8^ (syn5522790; Data Citation 1).

Somatic mutations were called from BAM files exported by the Dragen alignment using the Java version of VarDict^9^ in paired mode with an allele frequency threshold of 0.01. VarDict was able to recall germ line variants and also identify somatic variants that were present in the tumour samples but absent in the matched PBMC samples for each patient (syn6022465 and syn6022474; Data Citation 1).

### Copy number analysis

Genomic DNA (gDNA) was extracted as described above, but required two hundred and fifty nanograms of gDNA for downstream SNP array applications.

SNP array sample prep was done using the HumanOmni2.5–8 (Illumina, Inc., San Diego, CA, USA) as per manufacturer's recommended protocol as described:

http://support.illumina.com/content/dam/illumina-marketing/documents/products/workflows/workflow_infinium.pdf

http://support.illumina.com/content/dam/illumina-support/documents/documentation/chemistry_documentation/infinium_assays/infinium_lcg_assay/infinium-lcg-assay-guide-15023139-d.pdf

Samples were analysed by GenomeStudio and exported to text (syn5004874; Data Citation 1).

### RNA sequencing

Total RNA containing both mRNA as well as microRNA fractions was extracted from the tissues using the miRNeasy mini kit (Qiagen, Valencia, CA, USA) according to the manufacturer's protocol. The final elution was performed in 30 μl of RNase-free sterile distilled water. The concentration and integrity of the extracted total RNA were estimated using the Qubit 2.0 Fluorometer (Invitrogen) and Agilent 2,100 Bioanalyzer (Applied Biosystems, Carlsbad, CA, USA), respectively. Five hundred nanograms of total RNA and a RIN of 7.0 or higher were required for downstream RNA-seq applications. Poly-adenylated RNAs were isolated using NEBNext Magnetic Oligo d(T)25 Beads. The NEBNext mRNA Library Prep Reagent Set for Illumina (New England BioLabs Inc., Ipswich, MA, USA) was then used to prepare individually bar-coded next-generation sequencing expression libraries as per manufacturer's recommended protocol. Library quality was assessed using the Qubit 2.0 Fluorometer, and the library concentration was estimated by utilizing a DNA 1,000 Chip on an Agilent 2,100 Bioanalyzer. Accurate quantification for sequencing applications was determined using the qPCR-based KAPA Biosystems Library Quantification Kit (Kapa Biosystems, Inc., Woburn, MA, USA). Each library was diluted to a final concentration of 12.5 nM and pooled in an equimolar ratio prior to clustering. Paired-end sequencing (25 million, 50-bp, paired-end reads) was performed using a 200 Cycle TruSeq SBS HS v4 Kit on an Illumina HiSeq2500 sequencer (Illumina, Inc., San Diego, CA, USA).

Post-processing of the sequencing reads from RNA-seq experiments for each sample was performed using HudsonAlpha’s unique in-house RNA-seq data analysis pipeline (syn6035832; Data Citation 1). Briefly, quality control checks on raw sequence data for each sample were performed using FastQC (Babraham Bioinformatics, Cambridge, UK). Raw reads were mapped to the reference human genome hg19 using TopHat v2.0 (ref. 10) with the -p 4 and -r210 arguments. The alignment metrics of the mapped reads were estimated using SAMtools^11^ (syn6022474; Data Citation 1).

Reads were quantified by both Cufflinks v0.9.3 (ref. 10) (syn5492805; Data Citation 1) and FeatureCounts^12^ (syn5493036; Data Citation 1).

### Code availability

All data is currently stored in the synapse web portal, and is accessible using code in the Cutaneous NF Github repository at http://www.github.com/Sage-Bionetworks/dermalNF.

## Data Records

Data collected for each patient and sample have been annotated in the Synapse Table located within the online repository. Individual patients are described in Table 1, and the data available for each are described in Table 2.

## Technical Validation

Validation for each dataset was performed individually.

### SNP array data

Omni Array data were processed using Illumina Genome Studio and exported for further analysis using R to cluster regions that exhibited copy number alterations in either the germline (PBMC) or tissue samples.

**Figure 2:**
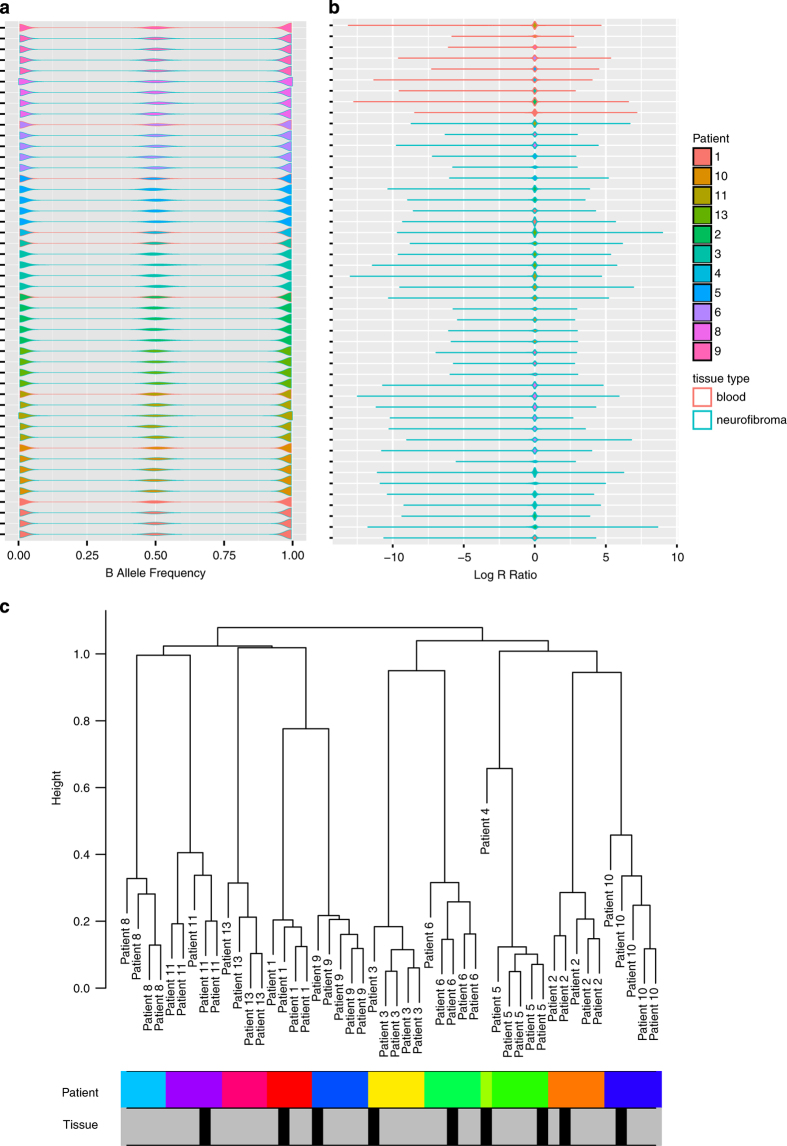
Copy number quality control metrics. Distribution of SNP array values across samples. Patient samples represented by different colours. Teal outline represents tumour samples while pink outline represents blood. (**a**) Represents B allele frequencies, (**b**) represents log R ratio values. (**c**) Clustering of segmented values, with rows below representing patient samples (colours) and tissue of origin (grey for tumour, black for blood).

To detect outliers we first plotted the logR ratio values and B allele frequencies computed by Genome Studio (Figs 2a,b) to insure that they follow a similar distribution across all samples. We then used hierarchical clustering to identify any possible outliers in the data. Specifically we clustered the median value of each segment of copy number alteration computed by the DNAcopy R package^13^. The resulting clusters, depicted in Fig. 2c, show strong corresponding clustering by patient.

### Whole genome sequencing

**Figure 3:**
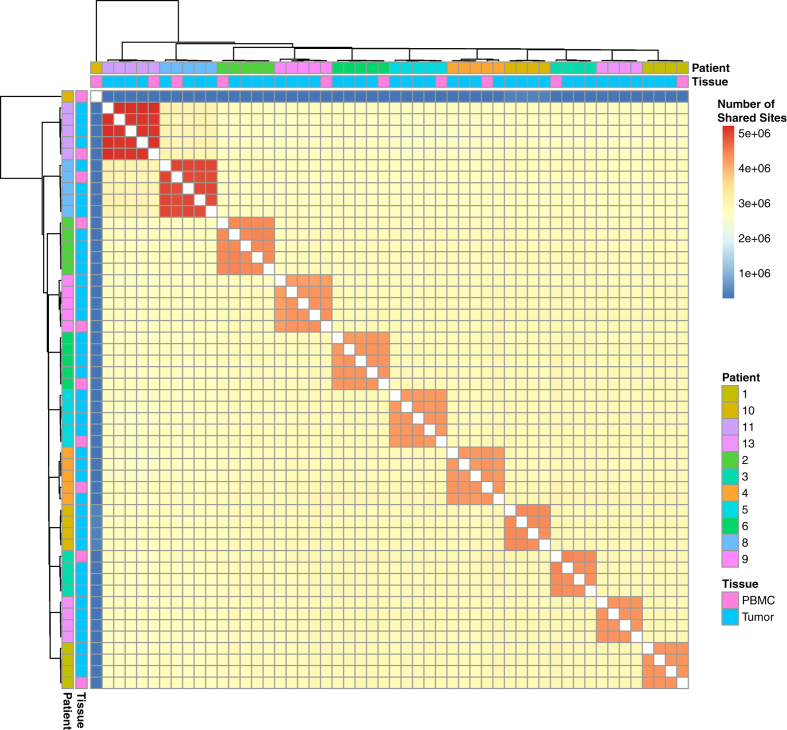
Number of genomic variant sites shared between all pairs of samples. Samples are labelled by patient and tissue type and indicate that all samples aside from the outlier (Patient 10 blood) cluster by patient.

In addition to basic library quality described above, we used the bcftools package^14^ to measure cross-sample discordance and identify potential outliers. Between each pair of samples we measured the number of shared variants called and the discordance measured between the pair of samples. The resulting values are depicted in Fig. 3. Similar to the copy number data patient, samples clustered with one another, with the exception of one sample, derived from Patient 10 PBMC, which was dropped from further analysis.

### RNA-Seq data

Quality control was performed on the RNA libraries prior to sequencing using the Agilent Bioanlyzer. Thirteen of the 44 samples were not sequenced as their RIN levels were less than 7. Final quality control of the samples was performed using the LabChip GX. Full QC and alignment statistics are shown in Table 3.

**Figure 4:**
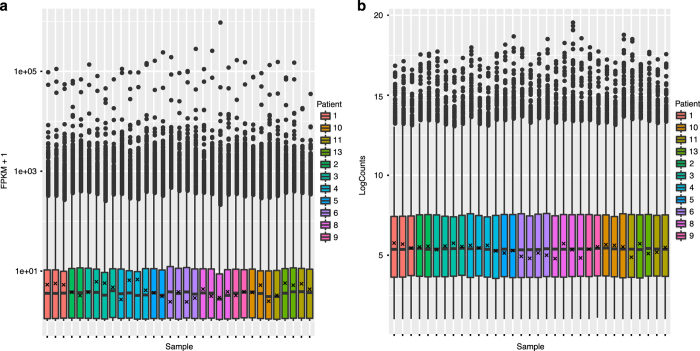
RNA-Seq count distribution. Distribution of RNA-Seq read counts per gene for both (**a**) DESeq normalized per-gene counts and (**b**) FPKM calculated by Cufflinks. Reads are distributed similarly across samples after filtering for unexpressed genes (<2 counts or FPKM of 0.1). Expression of *NF1* is indicated by ‘x’ in each sample.

After sequencing, RNA reads were aligned to Hg19 using TopHat v2.0 (ref. 10) and quantified using two distinct quantification methods: Cufflinks v0.9.3 (ref. 10) and FeatureCounts^12^. The numbers of normalized read counts (>2) that mapped to all transcripts are depicted in Figures and FPKM values (>0.1) are depicted in Fig. 4b.

## Usage Notes

All data are stored at the synapse web portal at http://www.synapse.org/cutaneousNF. Specific steps required to obtain access are described on the ‘Accessing the data’ wiki and involve:

1. Obtaining a Synapse account at http://www.synapse.org/register
2. Requesting access on the wiki and sending a brief email to CTF to describe how the data will be used

Inherent conditions for use of any data on Synapse are described on the Synapse governance site and apply to use of this dataset as well.

Scripts annotated to retrieve data from this repository can be found at http://github.com/Sage-Bionetworks/dermalNF.

## Additional Information

**How to cite this article:** Gosline, S. J. C. *et al.* A high-throughput molecular data resource for cutaneous neurofibromas. *Sci. Data* 4:170045 doi: 10.1038/sdata.2017.45 (2017).

**Publisher’s note:** Springer Nature remains neutral with regard to jurisdictional claims in published maps and institutional affiliations.

## Supplementary Material



## Figures and Tables

**Table 1:** Description of patients profiled in this study.

**Patient**	**Age**	**Gender**	**Race**	**Family history**	**Germline NF status**	**Number of Plexiforms**	**Number of Tumours profiled**
1	48	Female	White	Yes	SNV	0	6
2	34	Female	Asian	No	SNV	1–5	5
3	48	Female	Hispanic	No	SNV	0	5
4	36	Female	White	No	SNV	1	4
5	65	Male	White	No	SNV	0	5
6	46	Female	White	No	LOH	0	4
8	27	Female	Black	No	SNV	0	5
9	35	Male	White	No	SNV	0	4
10	58	Male	White	Yes	—	0	4
11	30	Female	Black	Yes	SNV	0	6
13	41	Female	White	No	—	0	4

**Table 2:** Description of tumours profiled in this study and the available data for each tumour.

**Patient**	**Tumour ID**	**Size (mm)**	**Whole genome Sequencing (WGS)**	**Single Nucleotide Polymorphism (SNP) array**	**RNA-Sequencing**
1	1	15			x
1	2	13			x
1	3	NA	x	x	
1	4	14			x
1	6	14	x	x	
1	9	15	x	x	
1	PBMC		x	x	
2	4	7	X	x	
2	6	6	X	x	
2	1	5	x	x	x
2	2	7			x
2	8	9	x	x	
2	PBMC		x	x	
3	1	11	x	x	x
3	2	16	x	x	x
3	3	20		x	x
3	4	15	x	x	x
3	PBMC		x	x	
4	1	8	x		x
4	10	7	x		x
4	4	5	x		x
4	9	10	x		x
4	PBMC		x	x	
5	11	10	x	x	
5	12	9	x	x	
5	13	11	x	x	
5	5	8	x	x	x
5	8	6			x
5	PBMC		x		
6	4	15	x	x	x
6	5	10	x	x	x
6	6	15	x	x	x
6	7	13	x	x	x
6	PBMC		x	x	
8	1	10			X
8	5	13	X	X	X
8	7	12	X	X	
8	4	10	X	X	x
8	6	10	X	X	X
8	PBMC		X		
9	1	12	X	x	X
9	6	5	X	X	x
9	7	5	X	X	
9	3	13	X	X	
9	PBMC		x	X	
10	1	11	x	x	x
10	2	20	x	x	x
10	3	10	x	x	x
10	4	5	x	x	
10	PBMC		x	x	
11	1	13	X	x	x
11	7	10	X	x	
11	8	10	X	x	
11	14	20			x
11	2	5	x	x	
11	3	3			x
11	PBMC		x	x	
13	1	18	x	x	x
13	2	7	x	x	
13	3	12	x	x	x
13	7	6	x	x	

**Table 3:** High throughput sequencing read statistics in patient tumours and associated peripheral blood mononuclear cells (PBMCs).

**Patient**	**Tumour**	**WGS Reads**	**Percent Aligned**	**RNA-Seq Reads**	**Percent RNA Aligned**	**RNA Integrity Number (RIN)**
1	1	NA	NA	2.60E+09	99.99	8.1
1	2	NA	NA	3.24E+09	99.99	7.6
1	3	8.00E+08	95.35	NA	NA	NA
1	4	NA	NA	2.69E+09	99.99	8.4
1	6	6.62E+08	94.84	NA	NA	NA
1	9	6.85E+08	96.08	NA	NA	NA
1	PBMC	8.02E+08	93.37	NA	NA	NA
2	1	7.82E+08	96.05	2.60E+09	99.99	7.3
2	2	NA	NA	2.52E+09	99.99	7.1
2	4	7.99E+08	96.47	NA	NA	NA
2	6	7.62E+08	96.74	NA	NA	NA
2	8	7.45E+08	95.14	NA	NA	NA
2	PBMC	7.56E+08	95	NA	NA	NA
3	1	7.33E+08	95.45	2.63E+09	99.99	6.9
3	2	7.42E+08	95.63	2.43E+09	99.99	7.6
3	3	7.99E+08	95.49	2.98E+09	99.99	7.3
3	4	8.93E+08	94.64	2.98E+09	99.99	8
3	PBMC	5.77E+08	94.82	NA	NA	NA
4	1	7.94E+08	92.44	2.67E+09	99.99	6
4	4	7.37E+08	94.5	2.99E+09	99.99	6.7
4	9	7.61E+08	94.46	3.93E+09	99.99	7.9
4	10	7.41E+08	93.64	3.06E+09	99.99	6.8
4	PBMC	9.84E+08	95.43	NA	NA	NA
5	5	7.67E+08	95.69	2.08E+09	99.99	8.7
5	8	NA	NA	2.28E+09	99.99	8
5	11	7.21E+08	94.52	NA	NA	NA
5	12	6.82E+08	93.94	NA	NA	NA
5	13	7.45E+08	93.73	NA	NA	NA
5	PBMC	5.71E+08	94.97	NA	NA	NA
6	4	7.35E+08	95.97	2.45E+09	99.99	8.3
6	5	5.42E+08	94.56	2.71E+09	99.99	9
6	6	7.72E+08	94.89	2.69E+09	99.99	6.3
6	7	6.83E+08	95.07	2.39E+09	99.99	8.8
6	PBMC	7.81E+08	95.46	NA	NA	NA
8	1	NA	NA	2.39E+09	99.99	8.8
8	4	6.89E+08	95.72	2.15E+09	99.99	8.1
8	5	8.10E+08	94.9	2.38E+09	99.99	7.9
8	6	7.05E+08	95.37	2.56E+09	99.99	9
8	7	7.00E+08	92.64	NA	NA	NA
8	PBMC	6.90E+08	93.4	NA	NA	NA
9	1	7.17E+08	95.31	3.35E+09	99.99	6.9
9	6	8.16E+08	94.77	2.82E+09	99.99	6.8
9	7	7.97E+08	91.96	NA	NA	NA
9	PBMC	6.30E+08	93.77	NA	NA	NA
10	1	7.43E+08	94.67	2.34E+09	99.99	7.5
10	2	7.39E+08	93.63	2.51E+09	99.99	6.5
10	3	8.20E+08	95.46	2.68E+09	99.99	6.4
10	4	8.07E+08	93.78	NA	NA	NA
10	PBMC	7.57E+08	94.48	NA	NA	NA
11	1	8.42E+08	96.04	NA	NA	NA
11	2	6.88E+08	95.64	NA	NA	NA
11	3	NA	NA	2.20E+09	99.99	6.6
11	7	1.24E+09	94.37	NA	NA	NA
11	8	5.81E+08	95.08	NA	NA	NA
11	14	NA	NA	NA	NA	NA
11	PBMC	7.14E+08	94.61	NA	NA	NA
13	1	7.44E+08	94.52	2.57E+09	99.99	6
13	2	7.97E+08	94.96	NA	NA	NA
13	3	8.06E+08	94.77	2.79E+09	99.99	8.2
13	7	9.34E+08	92.81	NA	NA	NA
